# Epstein-Barr virus-encoded RNAs (EBERs) complement the loss of Herpesvirus telomerase RNA (vTR) in virus-induced tumor formation

**DOI:** 10.1038/s41598-017-18638-7

**Published:** 2018-01-09

**Authors:** Ahmed Kheimar, Benedikt B. Kaufer

**Affiliations:** 1 Institut für Virologie, Freie Universität Berlin, Robert von Ostertag-Straße 7-13, 14163 Berlin, Germany; 2 Department of Poultry Diseases, Faculty of Veterinary Medicine, Sohag University, 82424 Sohag, Egypt

## Abstract

Marek’s disease virus (MDV) is an alphaherpesvirus that causes fatal lymphomas in chickens and is used as a natural virus-host model for herpesvirus-induced tumorigenesis. MDV encodes a telomerase RNA subunit (vTR) that is crucial for efficient MDV-induced lymphoma formation; however, the mechanism is not completely understood. Similarly, Epstein Barr-virus (EBV) encodes two RNAs (EBER-1 and EBER-2) that are highly expressed in EBV-induced tumor cells, however their role in tumorigenesis remains unclear. Intriguingly, vTR and EBER-1 have interaction partners in common that are highly conserved in humans and chickens. Therefore, we investigated if EBER-1 and/or EBER-2 can complement the loss of vTR in MDV-induced tumor formation. We first deleted vTR (v∆vTR) and replaced it by either EBER-1 or EBER-2 in the very virulent RB-1B strain. Insertion of either EBER-1 or EBER-2 did not affect MDV replication and their expression levels were comparable to vTR in wild type virus. Intriguingly, EBER-2 restored tumor formation of MDV that lacks vTR. EBER-1 partially restored MDV oncogenicity, while tumor formation was severely impaired in chickens infected with v∆vTR. Our data provides the first evidence that EBERs possess tumor-promoting properties in vivo using this natural model for herpesvirus-tumorigenesis.

## Introduction

Marek’s disease virus (MDV) is a highly oncogenic alphaherpesvirus that infects chickens and causes the most frequent clinically-diagnosed cancer in the animal kingdom^1,2^. Upon infection, MDV efficiently replicates in B cells and subsequently predominantly transforms CD4 T cells, resulting in deadly lymphomas^3,4^. Solid lymphomas can be detected in various visceral organs as early as 3 weeks post infection and in up to 100% of infected susceptible animals^1^. Several viral factors have been discovered that contribute to cancer formation including the major oncoprotein Meq (Marek’s EcoRI-Q-encoded protein)^5^, the viral interleukin-8 (vIL-8)^6,7^, MDV-encoded miRNAs^8,9^ and the virus encoded telomerase RNA (vTR)^10^.

vTR is dispensable for viral replication *in vitro* and *in vivo*, but is crucial for MDV-induced malignant transformation and is the most abundant viral transcripts in MDV-induced tumor cells^11^. It interacts with the chicken telomerase reverse transcriptase subunit (TERT) and enhances telomerase activity^10,12^. Intriguingly, MDV encoding a mutant vTR that does not mediate telomerase activity efficiently induced cancers as wild type virus^13^, demonstrating that the tumor-promoting functions of vTR are independent on its role of the telomerase complex^13^. vTR not only interacts with TERT but also with the ribosomal protein L22 (RpL22), a ribosomal protein that plays an important role in T-cell development^14,15^ and transformation. Although vTR has been shown to re-localize RpL22^13^, it remains unclear if this process contributes to cellular transformation.

Another viral RNA that binds and re-localizes RpL22 is the Epstein-Barr virus (EBV)-encoded RNA 1 (EBER-1). EBER-1 and the structurally related EBER-2 are highly expressed in EBV-latently infected^16^ and transformed cells^17^, however their role in transformation are still controversial. Deletion of both EBERs did not affect transformation of B cell *in vitro* ^18,19^, while others observed a role of EBER-2 in EBV-induced B cell proliferation^20^. In an EBV mouse model, deletion of the EBERs from the EBV genome did not change the viral persistence *in vivo* compared to wild type virus^21^; however, the tumor-promoting properties for EBERs were not assessed. Aside from RpL22, several factors have been shown to interact with EBER-1, including the Lupus erythematosis-associated antigen (La)^22^ and the double-stranded-RNA-activated protein kinase (PKR)^23,24^. EBER-2 also interacts with La^22^ as well as the transcription factor paired box protein 5 (PAX-5)^25^. Intriguingly, these factors are all conserved between humans and chickens.

In the current study, we investigated if EBER-1 and/or EBER-2 can complement the loss of vTR in MDV-induced tumor formation. We generated recombinant MDV-viruses that lack vTR and encode either EBER-1 or EBER-2 instead. Analysis of their replication properties *in vitro* and *in vivo* revealed that neither deletion of the entire vTR nor insertion of EBERs affects MDV replication. Deletion of the vTR severely impaired tumor formation. Intriguingly, expression of EBER-2 efficiently restored tumor formation, while EBER-1 only partially complemented the loss of vTR. Our study provides the first evidence that EBERs possess tumor-promoting effects *in vivo* using this natural animal model for herpesvirus-induced tumor formation.

## Results

### Generation and characterization of the recombinant viruses *in vitro*

**Figure 1:**
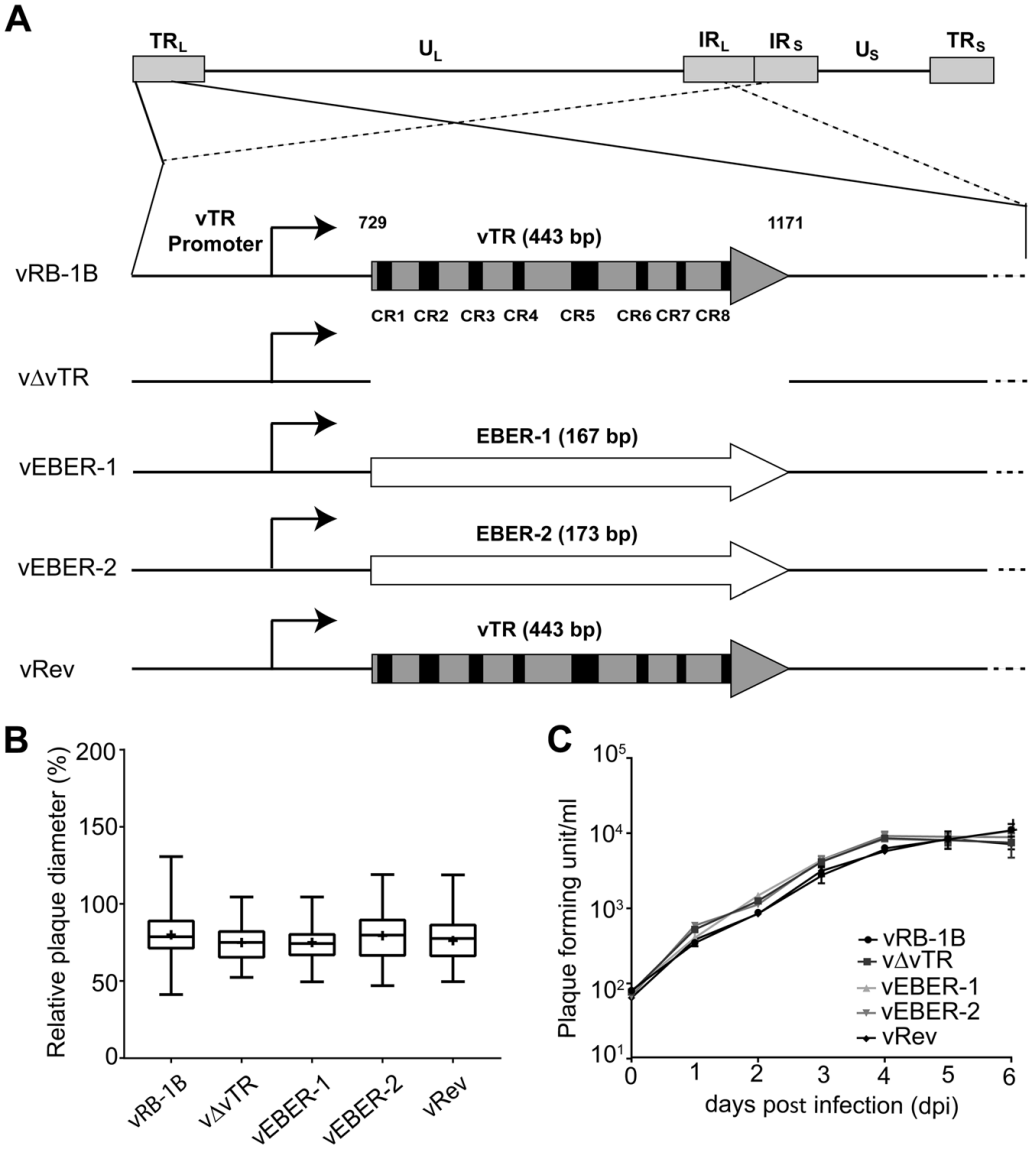
Generation and characterization of the recombinant MDV mutants. (**A**) Overview of MDV genome with a focus on the vTR with its eight conserved regions (CR1-CR8). Recombinant viruses that either lack the entire vTR (v∆vTR), harbor EBER-1 (vEBER-1) or EBER-2 (vEBER-2) instead of vTR are shown below. The vTR sequences were completely restored in the revertant virus (vRev). (**B**) Plaque size assays of indicated recombinant viruses. The plaque sizes are shown as box plots with minimums and maximums. Results are shown as the means of three independent experiments (p > 0.05; one-way ANOVA, n = 150). (**C**) Multi-step growth kinetics of indicated viruses. The average titer and standard deviations (error bar) are shown of triplicates of one independent experiment (p > 0.05; Kruskal-Wallis test).

To determine if EBERs can complement the loss of vTR, we generated recombinant viruses that encode either EBER-1 (vEBER-1) or EBER-2 (vEBER-2) instead of vTR (Fig. 1A). EBER-1 and EBER-2 were sequentially introduced into the RB-1B MDV strain lacking the entire vTR (vΔvTR) (Fig. 1A) using *en passant* mutagenesis. In addition, a revertant virus (vRev) was generated in which vTR was restored in the original locus. Mutants were analyzed by RFLP, Sanger and Illumina MiSeq sequencing (coverage > 1000-fold) to confirm that the entire virus genome is correct. To determine if deletion of vTR or insertion of EBERs affects viral replication, we assessed the replication of the recombinant viruses. Plaque size assays revealed that the recombinant viruses replicated comparable to wild type and revertant virus (Fig. 1B). We confirmed this observation using multi-step growth kinetics (Fig. 1C), highlighting that neither deletion of vTR nor insertion of EBERs alters MDV replication.

### Recombinant viruses efficiently express EBERs

**Figure 2:**
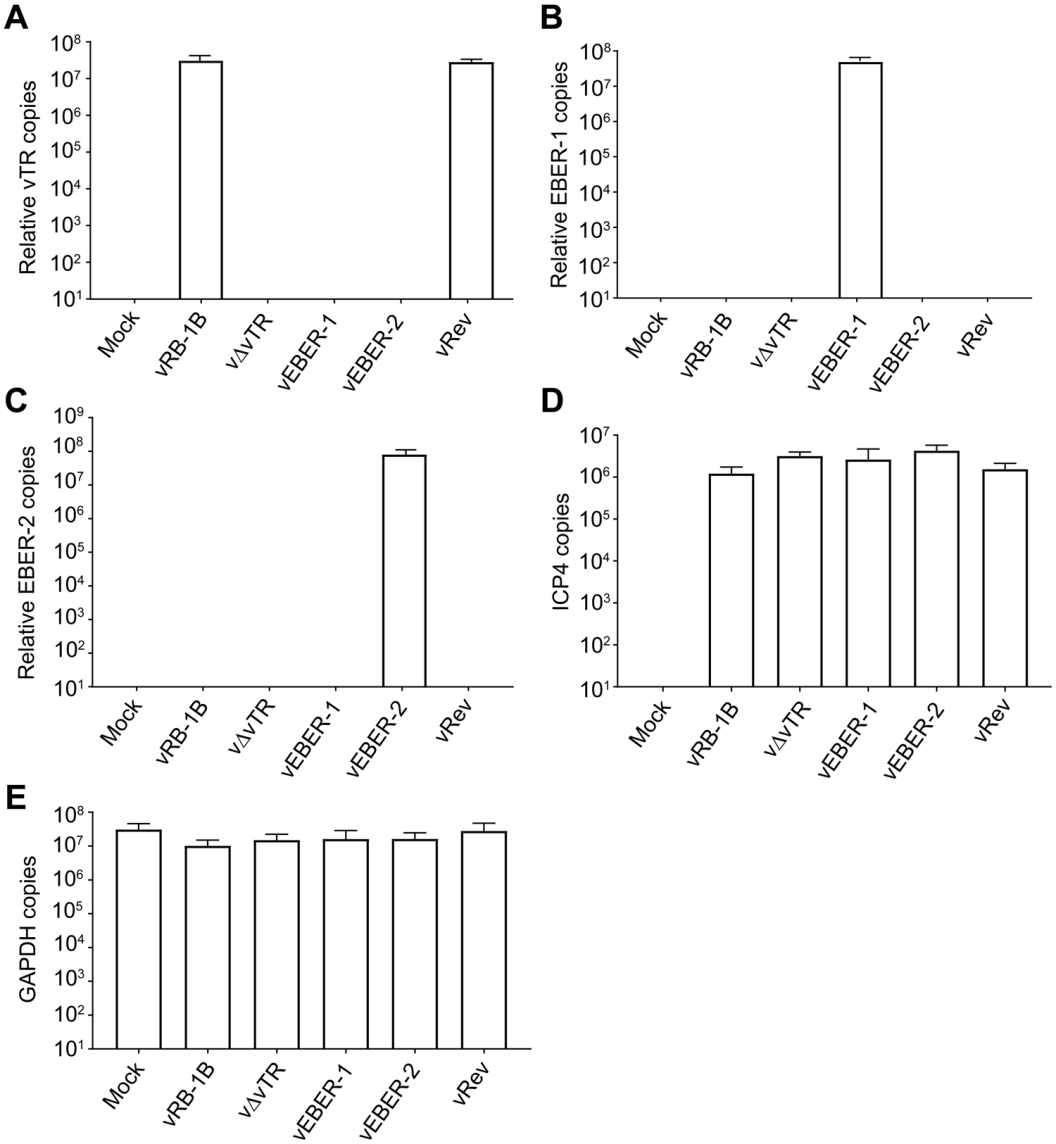
Quantification of vTR and EBERs expression in infected cells. One million CECs were infected with 1000 PFU of indicated viruses, RNA isolated 6 dpi and qRT-PCR performed. The mean copy numbers of (**A**) vTR, (**B**) EBER-1 and (**C**) EBER-2 is shown for indicated viruses relative to the expression levels of the cellular GAPDH and the viral ICP4 (p > 0.05; Kruskal-Wallis). Expression of (**D**) the viral ICP4 and (**E**) the cellular GAPDH control genes was not statistically different between the indicated viruses (p > 0.05; Kruskal-Wallis). Results are shown as means of three independent experiments with standard errors.

To determine if the EBERs are efficiently expressed during MDV replication, we infected CECs with the wild type or recombinant viruses and performed qRT-PCR. As expected, vTR was only expressed in wild type and revertant virus, while no vTR expression was detected upon deletion of the vTR gene (Fig. 2A). EBER-1 and EBER-2 were highly expressed in the corresponding recombinant viruses at copy numbers comparable to vTR in the wild type virus and revertant virus (Fig. 2B and C). No significant difference was observed for the expression of the viral ICP4 or the cellular GAPDH genes (Fig. 2D and E). Taken together, vEBER-1 and vEBER-2 efficiently expressed the expected EBER gene, while no vTR expression was detectable in both viruses.

### EBERs complement the loss of vTR in MDV-induced tumor formation

**Figure 3:**
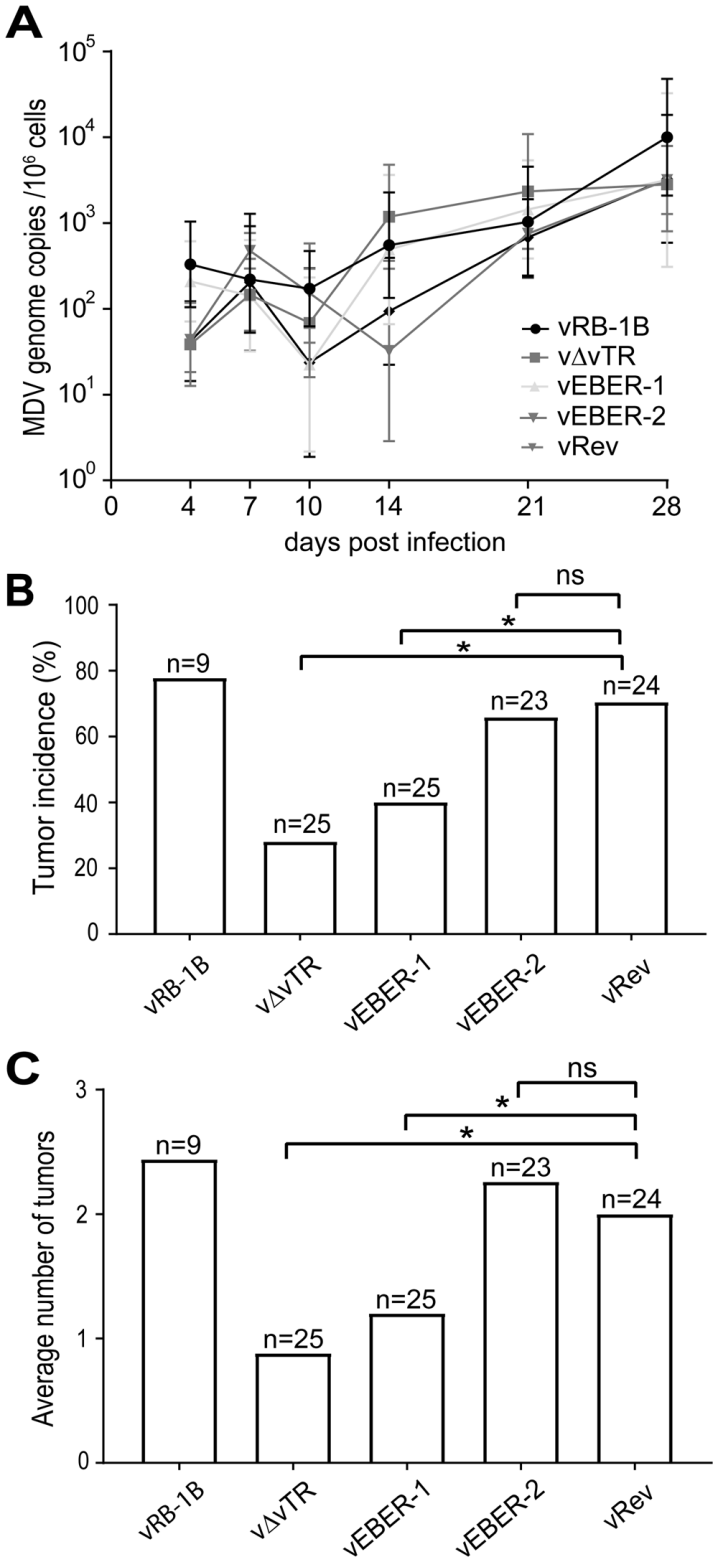
EBERs complement for the loss of vTR in MDV-induced tumor formation. (**A**) qPCR detecting MDV genome copies in the blood of chickens infected with vRB-1B (n = 9), v∆vTR (n = 25), vEBER-1 (n = 25), vEBER-2 (n = 23) or vRev (n = 24). Means MDV genome copies per million cells are shown for the indicated time points. (p > 0.05; Kruskal-Wallis test). (**B**) Tumor incidence in chickens infected with indicated viruses. Tumor incidence is shown in a percent of animals per group. Significant differences are indicated by an asterisk (p < 0.0125; Fisher’s exact test). (**C**) Mean number of gross tumors per animals infected with the indicated viruses, Significant differences are indicated by an asterisk (p < 0.0125; Fisher’s exact test).

To determine if EBERs can complement the loss of vTR in MDV-induced tumor formation, we infected one-day old chickens subcutaneously with 2,000 PFU of vRB-1B, v∆vTR, vEBER-1, vEBER-2 or vRev and monitored the onset of clinical symptoms and tumor formation. To investigate if the recombinant viruses replicated efficiently in infected animals, we quantified viral genome copies in the blood by qPCR. Replication of vΔvTR, vEBER-1 and EBER-2 was not significantly altered compared to the wild type and the revertant virus (Fig. 3A), indicating that expression of the EBERs did not affect MDV replication *in vivo*.

During the course of infection, animals were monitored for the development of clinical symptoms and tumors. In the absence of vTR, tumor incidence was significantly reduced (p < 0.0125) (Fig. 3B) as described previously^11^. Intriguingly, the tumor incidence of vEBER-2 was comparable to the wild type and revertant virus indicating that the expression of EBER-2 completely restored tumor formation. Expression of EBER-1 only partially restored MDV-induced tumor formation, as vEBER-1 (40%) only showed a mild increase in tumor incidence compared to vΔvTR (28%; Fig. 3B). To confirm that the EBERs are efficiently expressed in MDV-induced tumor cells, we quantified EBER-1 and EBER-2 expression in tumor tissue by qRT-PCR. Both EBER-1 and EBER-2 were highly expressed and at comparable levels (Supplementary Fig. 1).

To elucidate the effect of EBERs expression in tumor dissemination, we determined the numbers of organs with gross tumors during necropsy. The average number of the tumors per animals was significantly reduced in the absence of vTR compared to wild type and revertant virus (Fig. 3C). Most importantly, EBER-2 expression also efficiently restored tumor dissemination. No significant difference in the average number of tumors was observed between the animals infected with vEBER-2 compared to the wild type or revertant virus. A partial restoration was observed for vEBER-1 when compared to v∆vTR. Taken together, our data demonstrates that the EBV-encoded- EBERs can either fully (EBER-2) or partially (EBER-1) complement the loss of vTR in MDV-induced tumor formation using this small animal model for herpesvirus-induced oncogenesis. Our study provides thereby the first evidence that EBERs possess tumor promoting function *in vivo*.

## Discussion

vTR plays an important role in MDV-induced tumor formation, however the mechanism remains poorly understood. We recently demonstrated that vTR possesses tumor-promoting functions that are independent of its role in the telomerase activity^13^. The telomerase activity mediated by vTR only contributed to the rapid onset of tumors; however, tumor incidence and dissemination was not affected when incorporation of vTR into the telomerase complex was abrogated^13^. Therefore, vTR likely drives virus-induced transformation via the interaction with the ribosomal protein RpL22 and/or other cellular factors. Intriguingly, both vTR and EBER-1 interact and re-localize RpL22^13^, which is almost completely conserved between humans and chickens. Therefore, we set to determine if EBER-1 and/or EBER-2 can complement the loss of vTR in MDV-induced tumor formation.

We generated recombinant MDVs that express either EBER-1 or EBER-2 instead of vTR. Virus replication was not affected *in vitro* and *in vivo*, revealing that neither deletion of the entire vTR nor insertion of the EBERs affects MDV replication. Our data on the complete deletion of vTR is therefore consistent with the previously published partial deletions of the conserved regions (CR1-CR4) of vTR^11^. To confirm the efficient expression of EBER-1 and EBER-2, we performed qRT-PCR and we could demonstrate that EBERs were highly overexpressed. The observed expression levels of EBERs in MDV infected cells were also comparable to latently infected cells and EBV-induced cancers (>10^6^ per cell)^16,26^. EBER expression levels were also similar to vTR in wild type virus and revertant virus due to the strong nature of the vTR promoter^12^. Expression levels of vTR, and likely also the EBERs, play a crucial role in the transformation process as viruses that expressed vTR at lower levels were severely impaired in tumor formation *in vivo* ^27^.

To determine the effect of the complete deletion of vTR and if the EBERs can complement the loss of vTR, we infected SPF chickens with the recombinant viruses. As expected, deletion of the entire vTR severely attenuated MDV and is consistent with the partial deletion of the gene published previously^11^. Intriguingly, EBER-1 that also interacts and re-localizes RpL22 only partially restored MDV-induced tumor formation, suggesting that this interaction could indeed play a minor role in the cellular transformation. However, certainly also other interaction partners or mechanisms are responsible for vTR mediated tumor formation. Alternatively, the dysregulation of RpL22 could differ between EBER-1 and vTR, possibly due to differences in the binding affinity to the ribosomal protein as observed previously^11^. Surprisingly, EBER-2 expression efficiently restored MDV-induced tumor formation and metastasis of a virus that lacks vTR. Intriguingly, EBER-2 has been previously shown to inhibit apoptosis^28,29^ and increase cell-proliferation^30^, which could contribute to the increased tumor incidence of the EBER-2 expressing virus. It remains unknown which interaction partners of EBER-2 mediate these effects and if they are conserved between humans and chickens as La and PAX-5. We will address these aspects and if conserved stem loop structures in EBER-1, EBER-2 and vTR (Supplementary Fig. 2) mediate these functions in future studies.

Taken together, our data demonstrate that EBER-1 and EBER-2 possess tumor promoting activity that can complement the activity of vTR in MDV-induced transformation. Future studies will focus on the conserved interaction partners and possible mechanism(s) for EBER mediated transformation using this natural virus-host animal model for herpesvirus induced tumor formation.

## Methods

### Cells and viruses

Chicken embryo cells (CECs) were prepared from 11-day old Valo specific-pathogen free (SPF) embryos (ValoBioMedia) as described previously^31^. CECs were propagated in MEM supplemented with 10% FCS, 1% penicillin/streptomycin at 37 °C under a 5% CO_2_ atmosphere.

### Generation of recombinant viruses

Recombinant viruses encoding EBER-1 or EBER-2 instead of vTR were generated using a bacterial artificial chromosome (BAC) of the very virulent MDV strain RB-1B that lacks most of the internal repeat long region (IRL; pRB-1B∆IRL)^7^, which is rapidly restored upon virus reconstitution. Therefore, only one copy of vTR region had to be manipulated by two-step Red-mediated mutagenesis as described previously^32,33^, while the resulting recombinant virus contained the substitution/deletion in both loci^7^. First, we deleted the entire vTR, then sequentially introduced either EBER-1 (vEBER-1) or EBER-2 (vEBER-2) of the B95-8 EBV-strain (RefSeq M80517.1), allowing EBER expression under control of the native vTR promoter. In addition, a revertant virus (vRev) was generated in which the original vTR locus restored. Primers used for mutagenesis are listed in Table 1. Recombinant BAC clones were confirmed by RFLP, PCR and Sanger sequencing of the target area (Supplementary Fig. 3). In addition, we performed Illumina MiSeq sequencing to ensure that the entire nucleotide sequence of the constructs is correct. Recombinant viruses were reconstituted by transfection of CECs with BAC DNA as described previously^7,34^.

**Table 1:** Primers and probes for qRT-PCR, qPCR, DNA sequencing, and construction of the recombinant viruses.

Construct/target	Sequence (5′ → 3′)	
v∆vTR	For	CGGAGGAAGCTACAAGAGCCCCACGCGGGGTTCCCCCGGCGCGGCCCCGCGCGCACGACCT AGGGATAACAGGGTAATCGATTT
	Rev	TCTACTCACAGAGCCCCGCGCGCGGCTCAACGGCTCCAACGGTCGTGCGCGCGGGGCCGCGCCAGTGTTACAACCAATTAACC
vEBER-1	For	CGGAGGAAGCTACAAGAGCCCCACGCGGGGTTCCCCCGGCAGGACCTACGCTGCCCTAGA
	Rev	CGCGGCTCAACGGCTCCAACGGTCGTGCGCGCGGGGCCGCAAAACATGCGGACCACCAGC
vEBER-2	For	CGGAGGAAGCTACAAGAGCCCCACGCGGGGTTCCCCCGGCAGGACAGCCGTTGCCCTAGT
	Rev	GCGCGGCTCAACGGCTCCAACGGTCGTGCGCGCGGGGCCGCAAAAATAGCGGACAAGCCGA
vRev	For	CGGAGGAAGCTACAAGAGCCCCACGCGGGGTTCCCCCGGCACACGTGGCGGGTGGAAGGC
	Rev	ACGGCGTCGCTCCCACACGCGCGGCCCCGCGCGCACGACCGTTGGAGCCGTTGAGCCGCG
vTR locus	For	GCCCCTCTCTGCTCGCTCT
	Rev	TCCTGGCCTGGACGTGTG
vTR (qRT-PCR)	For	CCTAATCGGAGGTATT GATGGTACTG
	Rev	CCCTAGCCCGCTGAAAGTC
	Probe	FAM- CCCTCCGCCCGCTGTTTACTCG-TAM
EBER-1 (qRT-PCR)	For	GTGAGGACGGTGTCTGTGGTT
	Rev	TTGACCGAAGACGGCAGAA
	Probe	FAM- TCTTCCCAGACTCTGC-TAM
EBER-2 (qRT-PCR)	For	GCTACCGACCCGAGGTCAA
	Rev	GAGAATCCTGACTTGCAAATGCT
	Probe	FAM- AAGAGAGGCTTCCCGCC-TAM
ICP4 (qPCR)	For	CGTGTTTTCCGGCATGTG
	Rev	TCCCATACCAATCCTCATCCA
	Probe	FAM- CCCCCACCAGGTGCAGGCA-TAM
iNOS (qPCR)	For	GAGTGGTTTAAGGAGTTGGATCTGA
	Rev	TTCCAGACCTCCCACCTCAA
	Probe	FAM- CTCTGCCTGCTGTTGCCAACATGC-TAM
GAPDH (qRT-PCR)	For	GAAGCTTACTGGAATGGCTTTCC
	Rev	GGCAGGTCAGGTGAACAACA
	Probe	FAM- TGTGCCAACCCCCAAT-TAM

### Quantification of vTR and EBERs expression

vTR and EBER expression levels were determined *in vitro* and *in vivo* by qRT-PCR. Briefly, total RNA was extracted from viral infected CECs using the RNeasy Plus Mini Kit (Qiagen) and from tumor tissue using TRIzol Reagent (ThermoFischer) according to the manufacturer’s instruction. Samples were treated with DNase I (Promega) and cDNA generated using the high Capacity cDNA Reverse Transcription Kit (Applied Biosystems). vTR and EBER expression levels in the corresponding viruses were normalized to the expression levels of viral ICP4 and cellular GAPDH genes. Primers and probes used for qRT-PCR are shown in Table 1.

### Plaque size assays and growth kinetics

Virus replication and spread was determined by plaque size assays and multi-step growth kinetics as described previously^35^. For plaque size assays, at least 50 randomly selected plaques were captured and plaque areas were determined using Image J software (NIH). Significant difference in plaque diameters was evaluated by One-way analysis of variance (ANOVA).

### Ethics statement and *in vivo* experiments

All animal work was conducted according to relevant national and international guidelines for humane use of animals. Animal experiments were approved by the Landesamt für Gesundheit und Soziales (LAGeSo) in Berlin (approval number G0218/12). One-day old specific pathogen free (SPF) chickens (ValoBioMedia) were randomly assigned into four groups. Animals were infected subcutaneously with 2000 PFU of either wild type vRB-1B (n = 9), v∆vTR (n = 25), vEBER-1 (n = 25), vEBER-2 (n = 23) or the revertant virus vRev (n = 24). Peripheral blood samples were collected from the infected chickens at 4, 7, 10, 14, 21 and 28 dpi to determine MDV genome copy numbers in the blood, as described previously^34,36^. Chickens were monitored for clinical symptoms of MD on a daily basis throughout the 91 days of the experiment. To eliminate bias, the animal experiment was performed in a blinded manner until all data was collected and evaluated to avoid subjectivity. Animals were euthanized and examined for tumor lesions either once clinical symptoms were evident or after termination of the experiment. To confirm the presence of the introduced mutations in the virus genome, DNA was extracted from tumor tissue and the target region analyzed by Sanger sequencing.

### Quantification of MDV genome copies

DNA was extracted from the blood of the infected chickens using the E-Z96 blood DNA kit (OMEGA biotek, USA) following the manufacturer’s instructions. MDV genome copies were determined by quantitative PCR (qPCR) using specific primers and a probe for the MDV ICP4 gene (Table 1)^37,38^. ICP4 copy numbers were normalized to cellular inducible nitric oxide synthase (iNOS) gene as described previously^39^.

### Statistical analyses

The statistical analyses were performed using Graph-Pad Prism v7 and the SPSS software (SPSS, Inc). Plaque size assays were analyzed using one-way analysis of variance (ANOVA). MDV genome qPCR data were analyzed using the Kruskal-Wallis test. Data sets were first tested for normal distribution and results were considered significant when p < 0.05. Animal experiment data was analyzed by Fisher’s exact test, with a Bonferroni correction for multiple comparisons and results were considered significant when p < 0.0125.

## Electronic supplementary material

Supplementary Figure 1-3

## References

[1] Osterrieder N, Kamil JP, Schumacher D, Tischer BK, Trapp S (2006). Marek’s disease virus: from miasma to model. Nature reviews. Microbiology.

[2] Parcells, M. S., Burnside, J. & Morgan, R. W. In *Cancer Associated Viruses* (ed E. S. Robertson) 307–335 (Springer, science + business media, 2012).

[3] Calnek BW (2001). Pathogenesis of Marek’s disease virus infection. Current topics in microbiology and immunology.

[4] Davison, T. F., Nair, V. & Institute for Animal Health (Great Britain). *Marek’s disease: an evolving problem*. (Elsevier, 2004).

[5] Jones D, Lee L, Liu JL, Kung HJ, Tillotson JK (1992). Marek disease virus encodes a basic-leucine zipper gene resembling the fos/jun oncogenes that is highly expressed in lymphoblastoid tumors. Proceedings of the National Academy of Sciences of the United States of America.

[6] Parcells MS (2001). Marek’s disease virus (MDV) encodes an interleukin-8 homolog (vIL-8): characterization of the vIL-8 protein and a vIL-8 deletion mutant MDV. J Virol.

[7] Engel AT, Selvaraj RK, Kamil JP, Osterrieder N, Kaufer BB (2012). Marek’s disease viral interleukin-8 (vIL-8) promotes lymphoma formation through targeted recruitment of B-cells and CD4+ CD25+ T-cells. Journal of virology.

[8] Zhao Y (2011). Critical role of the virus-encoded microRNA-155 ortholog in the induction of Marek’s disease lymphomas. PLoS Pathog.

[9] Yao Y (2009). Differential expression of microRNAs in Marek’s disease virus-transformed T-lymphoma cell lines. The Journal of general virology.

[10] Fragnet L, Blasco MA, Klapper W, Rasschaert D (2003). The RNA subunit of telomerase is encoded by Marek’s disease virus. J Virol.

[11] Trapp S (2006). A virus-encoded telomerase RNA promotes malignant T cell lymphomagenesis. The Journal of experimental medicine.

[12] Kheimar, A., Previdelli, R. L., Wight, D. J. & Kaufer, B. B. Telomeres and Telomerase: Role in Marek’s Disease Virus Pathogenesis, Integration and Tumorigenesis. *Viruses* **9**, 10.3390/v9070173 (2017). 10.3390/v9070173 PMC5537665 28677643

[13] Kaufer BB, Arndt S, Trapp S, Osterrieder N, Jarosinski KW (2011). Herpesvirus Telomerase RNA (vTR) with a Mutated Template Sequence Abrogates Herpesvirus-Induced Lymphomagenesis. PLoS pathogens.

[14] Murre C (2007). Ribosomal proteins and the control of alphabeta T lineage development. Immunity.

[15] Anderson SJ (2007). Ablation of ribosomal protein L22 selectively impairs alphabeta T cell development by activation of a p53-dependent checkpoint. Immunity.

[16] Lerner MR, Andrews NC, Miller G, Steitz JA (1981). Two small RNAs encoded by Epstein-Barr virus and complexed with protein are precipitated by antibodies from patients with systemic lupus erythematosus. Proceedings of the National Academy of Sciences of the United States of America.

[17] Hanel P, Hummel M, Anagnostopoulos I, Stein H (2001). Analysis of single EBER-positive and negative tumour cells in EBV-harbouring B-cell non-Hodgkin lymphomas. The Journal of pathology.

[18] Kang MS, Kieff E (2015). Epstein-Barr virus latent genes. Experimental & molecular medicine.

[19] Swaminathan S, Tomkinson B, Kieff E (1991). Recombinant Epstein-Barr virus with small RNA (EBER) genes deleted transforms lymphocytes and replicates *in vitro*. Proceedings of the National Academy of Sciences.

[20] Wu Y, Maruo S, Yajima M, Kanda T, Takada K (2007). Epstein-Barr virus (EBV)-encoded RNA 2 (EBER2) but not EBER1 plays a critical role in EBV-induced B-cell growth transformation. Journal of virology.

[21] Gregorovic G (2015). Epstein-Barr Viruses (EBVs) Deficient in EBV-Encoded RNAs Have Higher Levels of Latent Membrane Protein 2 RNA Expression in Lymphoblastoid Cell Lines and Efficiently Establish Persistent Infections in Humanized Mice. Journal of virology.

[22] Tycowski KT (2015). Viral noncoding RNAs: more surprises. Genes & development.

[23] Gottlieb E, Steitz JA (1989). The RNA binding protein La influences both the accuracy and the efficiency of RNA polymerase III transcription *in vitro*. The EMBO journal.

[24] Takada KaN (2001). A. The role of EBERs in oncogenesis. Cancer Biol..

[25] Lee N, Yario TA, Gao JS, Steitz JA (2016). EBV noncoding RNA EBER2 interacts with host RNA-binding proteins to regulate viral gene expression. Proceedings of the National Academy of Sciences of the United States of America.

[26] Joab I (1999). Epstein-Barr virus and Burkitt’s lymphoma. Medecine tropicale: revue du Corps de sante colonial.

[27] Chbab N, Egerer A, Veiga I, Jarosinski KW, Osterrieder N (2010). Viral control of vTR expression is critical for efficient formation and dissemination of lymphoma induced by Marek’s disease virus (MDV). Veterinary research.

[28] Glickman JN, Howe JG, Steitz JA (1988). Structural analyses of EBER1 and EBER2 ribonucleoprotein particles present in Epstein-Barr virus-infected cells. Journal of virology.

[29] Weiss LM, Chen YY, Liu XF, Shibata D (1991). Epstein-Barr virus and Hodgkin’s disease. A correlative *in situ* hybridization and polymerase chain reaction study. The American journal of pathology.

[30] Laing KG (2002). *In vivo* effects of the Epstein-Barr virus small RNA EBER-1 on protein synthesis and cell growth regulation. Virology.

[31] Tischer BK, Smith GA, Osterrieder N (2010). En passant mutagenesis: a two step markerless red recombination system. Methods in molecular biology (Clifton, N.J.).

[32] Tischer BK, Kaufer BB (2012). Viral bacterial artificial chromosomes: generation, mutagenesis, and removal of mini-F sequences. Journal of biomedicine & biotechnology.

[33] Tischer BK, von Einem J, Kaufer B, Osterrieder N (2006). Two-step red-mediated recombination for versatile high-efficiency markerless DNA manipulation in Escherichia coli. BioTechniques.

[34] Jarosinski KW (2007). Horizontal transmission of Marek’s disease virus requires US2, the UL13 protein kinase, and gC. J Virol.

[35] Schumacher D, Tischer BK, Trapp S, Osterrieder N (2005). The protein encoded by the US3 orthologue of Marek’s disease virus is required for efficient de-envelopment of perinuclear virions and involved in actin stress fiber breakdown. J Virol.

[36] Jarosinski K, Kattenhorn L, Kaufer B, Ploegh H, Osterrieder N (2007). A herpesvirus ubiquitin-specific protease is critical for efficient T cell lymphoma formation. Proceedings of the National Academy of Sciences.

[37] Chang L-Y (1999). Telomerase activity and *in situ* telomerase RNA expression in oral carcinogenesis. Journal of Oral Pathology & Medicine.

[38] Jarosinski KW, Osterrieder N, Nair VK, Schat KA (2005). Attenuation of Marek’s disease virus by deletion of open reading frame RLORF4 but not RLORF5a. J. Virol.

[39] Kaufer BB, Jarosinski KW, Osterrieder N (2011). Herpesvirus telomeric repeats facilitate genomic integration into host telomeres and mobilization of viral DNA during reactivation. J Exp Med.

