# Supplementary material for: Epstein-Barr virus-encoded RNAs (EBERs) complement the loss of Herpesvirus telomerase RNA (vTR) in virus-induced tumor formation
Source: Sci Rep. 2018 Jan 9;8:209. doi: 10.1038/s41598-017-18638-7 (PMC5760702; doi:10.1038/s41598-017-18638-7)
Supplement: Supplementary file 1 — Supplementary Figure 1-3 [file 41598_2017_18638_MOESM1_ESM.pdf]

Epstein-Barr virus-encoded RNAs (EBERs) complement the loss of Herpesvirus telomerase RNA (vTR) in virus-induced tumor formation.

Ahmed Kheimar[1,2], Benedikt B. Kaufer[1]*

[1]Institut für Virologie, Freie Universität Berlin, Robert von Ostertag-Straße 7-13, 14163 Berlin, Germany;

[2]Department of Poultry Diseases, Faculty of Veterinary Medicine, Sohag University, 82424 Sohag, Egypt.

* **Corresponding author:**

Benedikt B. Kaufer, Institut für Virologie, Freie Universität Berlin, Robert von Ostertag-Straße 7-13, 14163 Berlin, Germany.

Phone: +49-30-838-51936, Fax: +49-30-838-51847, e-mail: b.kaufer@fu-berlin.de

Supplementary Figure 1. Quantification of EBERs in tumor tissues. To confirm EBER-1 and EBER-2 expression in MDV-tumors, total RNA isolated from tumor tissue and qRT-PCR performed. Mean copy numbers of A) EBER-1 and B) EBER-2 are shown for tumor tissues derived from animals infected with indicated viruses and were normalized to cellular GAPDH. Results are shown as means of three tumor tissues for each group with standard errors (error bars).

Supplementary Figure 2. Secondary structure prediction of vTR, EBER-1 and EBER-2. The predicted secondary structure of vTR, EBER-1 and EBER-2 with conserved stem-loop structures highlighted (dashed boxes), based on previous publications[1-3].

Supplementary Figure 3. Confirmation of EBER sequences in MDV-induced tumors. DNA was extracted from tumor tissue and the target region in the virus genome containing either EBER-1 or EBER-2 was analyzed by Sanger sequencing. Alignment of A) EBER-1 and B) EBER-2 from the corresponding tumor tissue with the reference sequence is shown. Identical sequences are represented by dots.

Supplementary Figure 1:

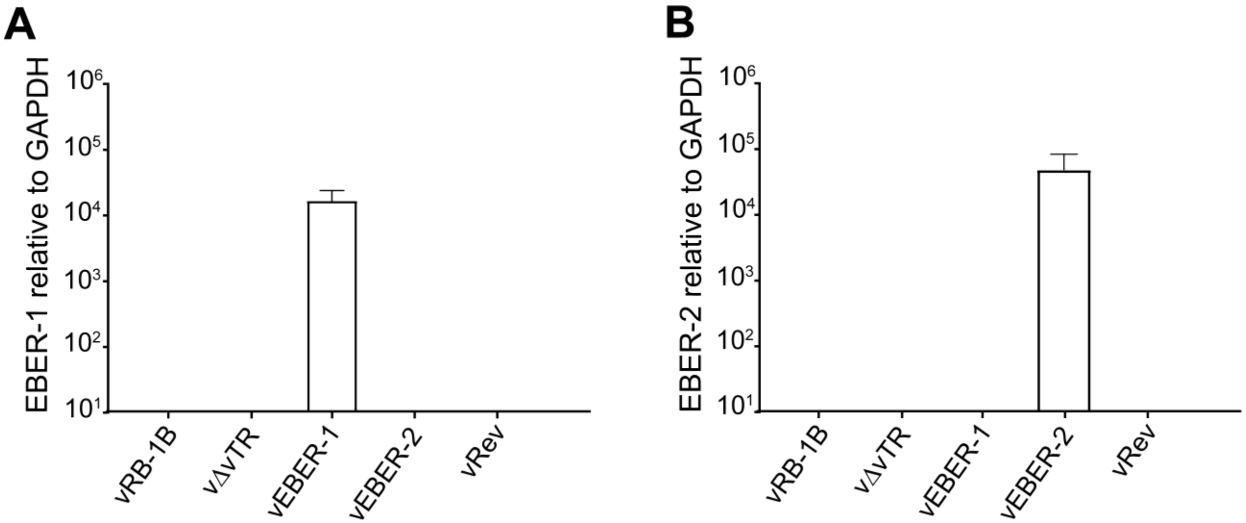

Supplementary Figure 2:

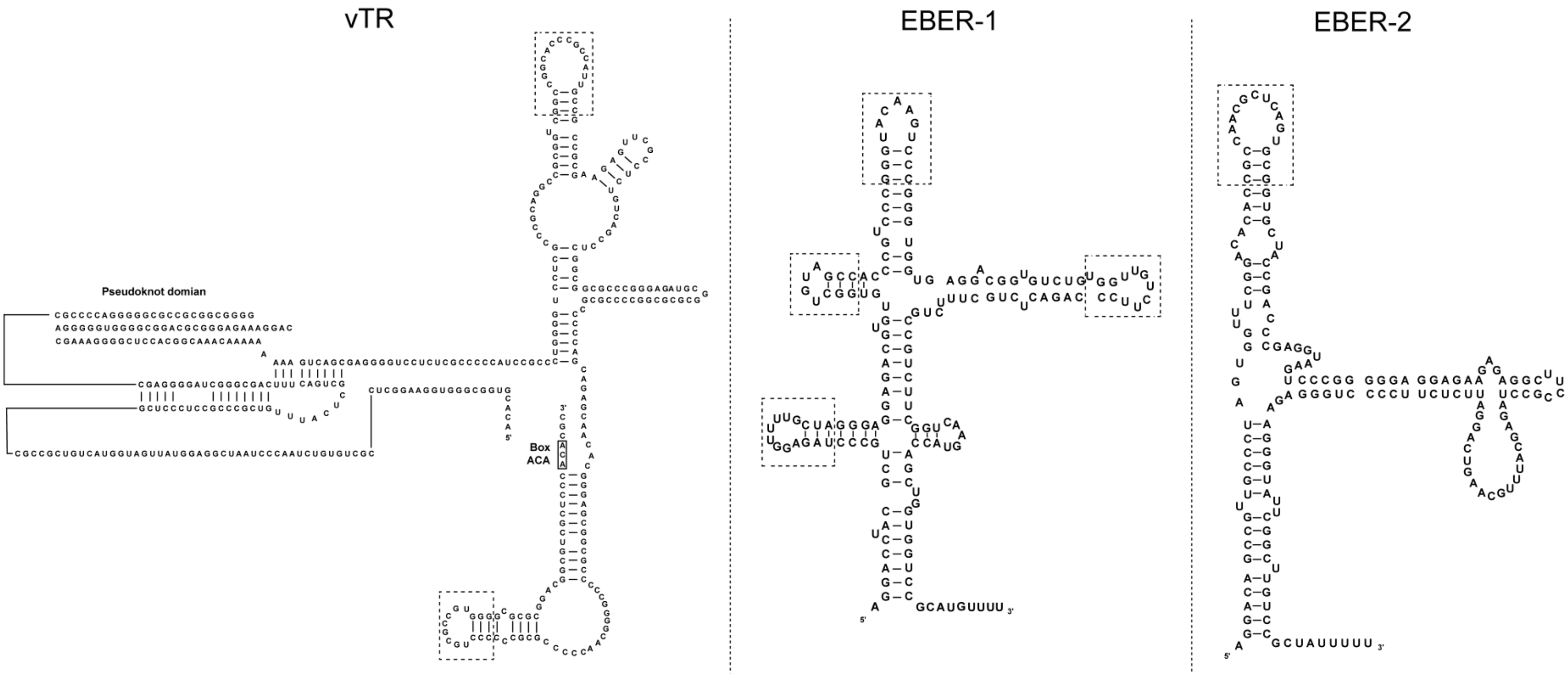

Supplementary Figure 3:

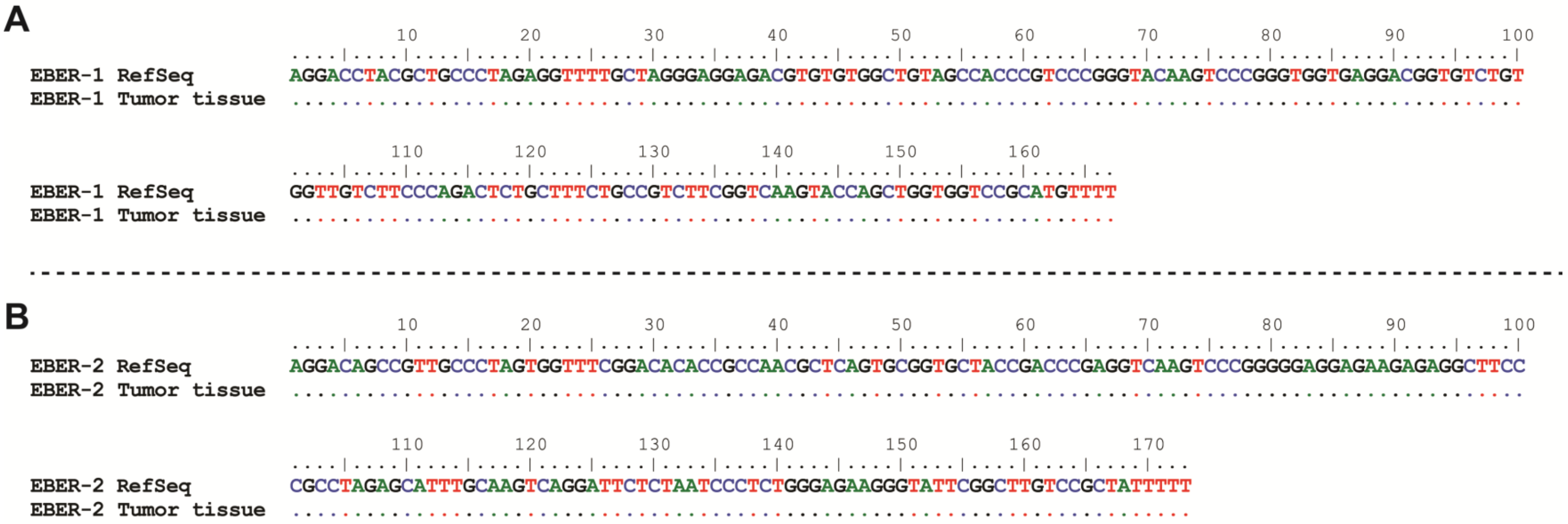

References:

1 Dobbelstein, M. & Shenk, T. In vitro selection of RNA ligands for the ribosomal L22 protein associated with Epstein-Barr virus-expressed RNA by using randomized and cDNA-derived RNA libraries. *J Virol* **69**, 8027-8034 (1995).

2 Fragnet, L., Blasco, M. A., Klapper, W. & Rasschaert, D. The RNA subunit of telomerase is encoded by Marek's disease virus. *J Virol* **77**, 5985-5996 (2003).

3 Tycowski, K. T. *et al.* Viral noncoding RNAs: more surprises. *Genes & development* **29**, 567-584, doi:10.1101/gad.259077.115 (2015).
